# Assessing red deer hunting management in the Iberian Peninsula: the importance of longitudinal studies

**DOI:** 10.7717/peerj.10872

**Published:** 2021-02-05

**Authors:** Antonio José Carpio Camargo, Jose Barasona, Pelayo Acevedo, Yolanda Fierro, Christian Gortazar, Carlos Vigal, Ángel Moreno, Joaquin Vicente

**Affiliations:** 1 Department of Zoology, University of Córdoba, Córdoba, Spain; 2 SaBio, Instituto de Investigación en Recursos Cinegéticos, IREC (CSIC-UCLM-JCCM), Ciudad Real, Spain; 3 VISAVET Health Surveillance Centre, Department of Animal Health, Veterinary School, Complutense University of Madrid, Madrid, Spain; 4 Yolfi Properties, Ciudad Real, España; 5 Los Quintos de Mora, Organismo Autónomo de Parques Nacionales, Toledo, Spain

**Keywords:** Artificial feeding, *Cervus elaphus*, Management, Population dynamics, Population growth, Recruitment rate

## Abstract

Understanding the dynamics of a wildlife population in relation to hunting strategies is essential to achieve sustainable management. We used monitoring data over 25 years from two red deer (*Cervus elaphus*) populations with different management (with and without supplemental feeding) in South Central Spain to: (i) characterise the density dependence of population dynamics under contrasted management, and (ii) provide the basis for sustainable extraction by considering the theoretical maximum sustainable yield (MSYt) as the reference. The red deer population displayed a typical management reactive culling approach (‘saw-tooth-like’ curves), with occasional strong annual harvests but not occurring on a regular basis. Interestingly, we found reduced population growth at high densities in both populations, indicating that density-mediated factors determined population growth even when artificial feeding was provided. However, no effects of sex not age class of the extracted population on the population growth rate were determined. The total number of animals hunted was only slightly above those predicted by MSYt (i.e. *K*_50%_) in both populations, despite high densities close to theoretical *K*, being consistent throughout the study period. The extraction rates (30.3 and 34.0%, for supplemented and unsupplemented populations, respectively) were 13.3% and 10.2% lower compared to the MSYt situation in the unsupplemented and supplemented populations, respectively. Long term population monitoring data provided feasible and suitable baseline values to optimise the sustainable exploitation of red deer populations in the Mediterranean ecosystem under these contrasting management scenarios. Adaptive management, involving objective-driven decision making informed by data on red deer population dynamic, can contribute (i) to maximising the total extraction over the long term while (ii) reducing the ecological impact of high population densities.

## Introduction

The populations of deer species have increased and expanded throughout Europe and North America over the last century (Apollonio, Andersen & Putman, 2010; Mysterud, 2010; VerCauteren et al., 2011) due to several factors, among which the change in management (e.g. use the quotas, hunting legislation), has been highlighted as a key factor (Acevedo & Cassinello, 2009; Putman, Apollonio & Andersen, 2011; Carpio et al., 2017). The management of deer has become a focus of research with the recent large increases in population size (Skonhoft et al., 2013; Martínez & Martín, 2017). Currently, the red deer (*Cervus elaphus*) population sizes in numerous areas are above the biological and humans dimensions of carrying capacities (Carpenter, Decker & Lipscomb, 2000) and therefore undesirable ecological situations and social conflicts are becoming more frequent (Perea, Girardello & San Miguel, 2014; Vicente et al., 2019; Martínez-Jaúregui et al., 2020). In a global scenario in which naturally occurring large predators are scarce, the regulation of populations by hunting is one of the most relevant driver of deer population dynamics (Milner et al., 2006). However, density-dependence, which depends on intraspecific competition together with environmental stochasticity (e.g. drought, heavy snow and other non-seasonal processes), are natural determinants that also affect ungulate population dynamics that can alter the management goals and therefore the hunting rents (Sæther, 1997; Torres-Porras, Carranza & Perez-Gonzalez, 2009; Rodriguez-Hidalgo et al., 2010).

The successful management of wildlife populations—with ecological and/or economical goals—requires a clear understanding of the processes involved in population dynamics from a long-term perspective (Gaillard, Festa-Bianchet & Yoccoz, 1998). The recruitment rate expresses the effect of a broad set of extrinsic and intrinsic factors on population dynamics (Hamel, Côté & Festa-Bianchet, 2010). The number of deer harvested annually should be based on the population recruitment (i.e. the number of surviving calves born the previous year) and the residual population from the previous hunting season (Skonhoft et al., 2013). Much of the theory underlying harvest management, including the concept of density dependence, is based on carrying capacity (Martínez & Martín, 2017; Kapota & Saltz, 2018). According to theoretical population growth models (Schoener, 1973), the highest levels of recruitment rate are reached at medium population densities (close to 50–60% of the carrying capacity for red deer; Buckland et al. (1996) for adult stags; Mandujano (2007) for white-tailed deer; DeCalesta & Eckley (2019)), since density-dependent mechanisms that operate mainly through female body condition and fertility apparently peak after these densities limit further population growth (Borowik, Wawrzyniak & Jędrzejewska, 2016). There is an ideal point on the curve of density dependence where the harvest rate is maximised (maximum sustainable yield, MSY), that is the largest yield that can be taken from a population over an indefinite period. MSY is an interesting reference point for comparisons (for instance, within a population through time) because it meets the requirements of maximising hunting.

The Iberian red deer (*C. e. hispanicus*) is one of the most important game species in the Iberian Peninsula, particularly in the central and southern regions, where big game hunting is a relevant socio-economic activity (Fierro et al., 2002; Carranza, 2010; Vingada et al., 2010). The prevailing management systems in these areas often involve fencing, artificial feeding and translocations (Rodriguez-Hidalgo et al., 2010; Martínez-Jaúregui & Herruzo, 2014). Current local densities may reach over 40 ind/km^2^ (Vicente et al., 2007; Acevedo et al., 2008), which might lead to unsustainable situations, triggering negative effects at different levels of the ecosystem (Gortázar et al., 2006; Carpio et al., 2014a, 2014b; Perea, Girardello & San Miguel, 2014). There is a need to understand the population dynamics of red deer in Mediterranean areas in order to establish sustainable management plans based on scientific knowledge and that are compatible with maximising hunting as a relevant socio-economic activity while impeding the negative impacts of overabundance. We used monitoring data from over 25 years in 2 red deer populations in South Central Spain with different management systems (with and without supplemental feeding) to: (i) characterise the density dependence of population dynamics under contrasted management, and (ii) provide the basis for sustainable extraction, considering the population-specific theoretical maximum sustainable yield (MSYt) as a practical reference for comparison purposes.

## Materials and Methods

### Study area

The two study populations are located in Castilla-La Mancha, South Central Spain. The Mediterranean woodlands and scrublands in the area constitute a continuous distribution of independently managed private or public hunting estates, including natural reserves. One of the estates is a private property devoted to game hunting (LM; 38°55′N, 0°36′E; 600–850 m a.s.l.) and the other is a public hunting preserve (LQ; 39°45′N, 4°15′E; 600–1,100 m a.s.l.). Since 1989, the LM deer population has received supplementary forage (protein-rich pellets) all the year-round, and in summer, alfalfa (*Medicago sativa*) has been supplied on the ground. The deer population in the LQ estate did not receive supplementary forage. They were selected as being representative of the more frequent typologies of big-game hunting estates in Mediterranean Spain. Table 1 summarises the main characteristics of the study areas.

**Table 1:** Characteristics of the two red deer populations (mean values ± 95% IC SD) for the complete temporal series under both types of management systems (LQ, without supplemental feeding; LM, with supplemental feeding).

Population parameter	LM	LQ
Surface (ha)	868	6862
Density (deer/km^2^)	37.8 ± 14.8	32.9 ± 10.3
Sex-ratio (n° females/n° males)	1.45 ± 1.84	1.35 ± 0.49
Birth rate (n° calves/n° females)	0.63 ± 0.19	0.48 ± 0.09
Survival rate (n° yearlings/n° of calves from previous year) * 100	95.2 ± 56	58.9 ± 39.9
% Annual harvest (% of harvested population)	23.8 ± 16.8	17.5 ± 16.2
Harvest density (n° deer hunted/km^2^)	9.1 ± 7.5	5.6 ± 4.7
Recruitment rate (*N*_1_,_t+1_/*N*_≥1_,_t_)	0.34 ± 0.22	0.16 ± 0.12
% calves	25.1 ± 6.6	21.1 ± 4.1
% females	40.4 ± 8.2	44.2 ± 5.4
% yearlings	23.9 ± 10.2	12.3 ± 6.4
% adults	62.9 ± 7.5	72.8 ± 5.6
Growth rate (*N*_≥1,t+1_/*N*_≥1,t_)	1.06 ± 0.43	1.00 ± 0.41

The Mediterranean habitat is characterised by savannah-like landscape called ‘dehesas’, with evergreen oak, *Quercus ilex*, woodlands, and scrublands (dominated by *Cystus* spp., *Erica* spp., *Pistacia* spp., *Phyllirea* spp. and *Rosmarinus* spp.), scattered pastures and small areas of crops.

### Estimation of red deer density and population growth rates

Between 1989 and 2015, the annual harvest rates and deer population sizes were estimated at both hunting estates. The deer density was estimated in the LQ estate by spotlight censuses from a 4 × 4 vehicle that were performed during August–September. Each transect was an average of 20.3 km ± 2.34 (S.E.) in length, and the speed of the vehicle was <15 km/h. The distance between the observer and the deer was measured with a Leica LRF 1200 Scan telemeter (Solms, Germany) (range 15–1,100 m; precision ±1 m/±0.1%). The annual abundance of the deer was estimated using the distance sampling method (Buckland et al., 2004) with Distance 6.0 software (Thomas et al., 2010). Half-normal, uniform and hazard rate distributions for the detection function were fitted against the data using cosine, Hermite polynomial and simple polynomial adjustment terms, which were fitted sequentially. The selection of the best model and adjustment term were based on Akaike’s Information Criterion (AIC). More details on the analytical framework can be found in Acevedo et al. (2008).

The size of the deer population at the LM estate (Table 1) was estimated annually by using repeated direct counts at feeding sites at the end of July and during the rutting season (total = 8 days/year), that is, September and October (see Rodriguez-Hidalgo et al., 2010). In the summer, most deer congregate around feeding sites and are counted at dusk. The estimates obtained from direct counts are equivalent to those obtained using the same approach used in LQ, that is spotlight counts and distance sampling. We evaluated the consistence between distance sampling and direct counts at feeding sites in three different years in LM (2003, 2006 and 2020). The results showed a high equivalence in the estimates obtained; 0.32 (CV 16.8%)–0.36 deer/km^2^ (distance sampling—direct counts), 0.41 (CV 24.1%)–0.42 deer/km^2^ and 0.41 (CV 20.4%)–0.48 deer/km^2^ for 2003, 2006 and 2020, respectively.

We obtained the sex- and age-specific structure of the deer populations during 1989–2015 period using the number of animals recorded for each sex and age during the transects (LQ) and direct counts (LM). We registered the number of calves (<1 year old), yearlings (between 1 and 2 years) and adults (>3 years) (Landete-Castillejos et al., 2004). We combined the numbers for both sexes and defined the sum of yearlings plus adults as the total population size in year t (*N*_≥1,_*_t_*). We defined the number of yearlings as the number of recruits at the end of the year. We calculated the adult sex/ratio (females/males), the birth rate (calves/females), the survival rate (yearlings/calves from the previous year)*100, the recruitment rate in year t: (*N*_1_,_*t*+1_/*N*_≥1_,*_t_*) (Ueno, Kaji & Saitoh, 2010) and the exponential population growth in year *t* (ln(*N*_*t*+1_/*N_t_*)) (Hebblewhite, Pletscher & Paquet, 2002); where *N_t_* represents the population of a given year and *N*_*t*+1_ is the population of the next year to characterise the population dynamics. Henceforth, we refer to the exponential population growth rate as the population annual growth rate. We specify that our study was based on the logistic population growth model (which is not linear). However, as abovementioned, ln(*N*_*t*+1_/*N_t_*) (i.e. Exponential growth rate or intrinsic rate of increase) is a linear function of N (Hebblewhite, Pletscher & Paquet, 2002). The intrinsic rate of increase, as defined in the exponential equation, is not a constant number at all but rather is itself a function of the density of the population, and it can be assumed a linear approximation.

### Data analysis: theoretical maximum sustainable yields harvest (MSYt)

Ecological carrying capacity can be approached by the number of individuals that the environment ‘can support’ in a given area (Chapman & Byron, 2018). Therefore, in this study the ecological carrying capacity, represented by the *K* of the logistic equation, is useful to study the balance between forage resources, negative impacts and population productivity. The theoretical Maximum Sustainable Yields harvest (MSY) is the maximum number of deer that can be harvested from the population on a continual basis (McCullough, 1992), in this case, based on the logistic population growth model. Theoretically, and to establish a reference practical point for internal comparisons in our study populations, we considered that the population level that produces the MSY is approximately 50% of *K* (Jensen, 1996), which has been referred to as the Economic Carrying Capacity (Caughley, 1979), or I-Carrying Capacity (i.e. *K*/2 or population abundance at MSY, MacNab, 1985; McCullough, 1992; Buckland et al., 1996). When the growth rate (as density-dependence relationship) is modelled by logistic equation, *r*_0_ is the maximal rate of population growth from low density and *K* is indicated where the regression cuts the *X* axis (i.e. exponential population growth is 0). Under the logistic model, a negative linear relationship between the growth rate and density (including confidence intervals) is expected, which is used as a simple test of statistical density dependence (Wolda & Dennis, 1993; Reynolds & Freckleton, 2005; Lebreton & Gimenez, 2013).

A linear model was used to evaluate the effects of density on the population dynamics and calculate the related parameters. Beyond analysing the complete set of potential factors determining the growth rate (the authors, in preparation) our model included as covariables the age and sex structure of the harvest. Age and sex structure are critical factors regarding the demographic parameters affected by population density or supplementary feeding, and they are quite relevant in determining culling strategies (Trenkel, 2001). For this purpose, age class was simplified to individuals <1 year/(calves) and >1 year (broadly, adults). These extractions, separately by sex and ages, are expressed in terms of proportion of individual extracted respect to available numbers. In order to determine the potential impacts of factors depending on the management regime (supplement vs unsupplemented), the population density and their respective interactions with other explanatory factors were included in the model.

The fitted linear regression on the exponential growth rate on the density helped us to calculate the theoretical *K* parameter, that is the size of the population for which the population growth rate is 0. The residual population accommodating MSY was also calculated as a proxy to a sustainable extraction of the populations (because it meets the requirements of maximising hunting while reducing the impacts of overabundance). According to the logistic model, at low densities, the population growth rate is the maximum, but the product (*r*N = net recruitment (number of net individuals that is added to the population per unit of time), *r* is referred to as the intrinsic rate of increase) is low. As density increases, the growth rate decreases and *N* increases, the net recruitment increases to a maximum *K*/2. This point, the MSY, is the theoretical maximum number of individuals that can be extracted from the population on the long-term. Since this theoretical parameter was considered a proxy for purposes of comparison between the populations in our illustrative scenario, following Stephens & Sutherland (1999) we were conservative in calculating and establishing a safety margin to avoid overexploitation (data for deer in the literature approaches 60% of *K*) (Clutton-Brock et al., 2002; Kaji et al., 2010). The calculation of such parameters allows us to compare real population extraction rates and those based on the assumption of the MSY value over years in two sets of contrasting data under different management regimes.

## Results

### Characterization of red deer population dynamics in the contrasted study populations

**Figure 1:**
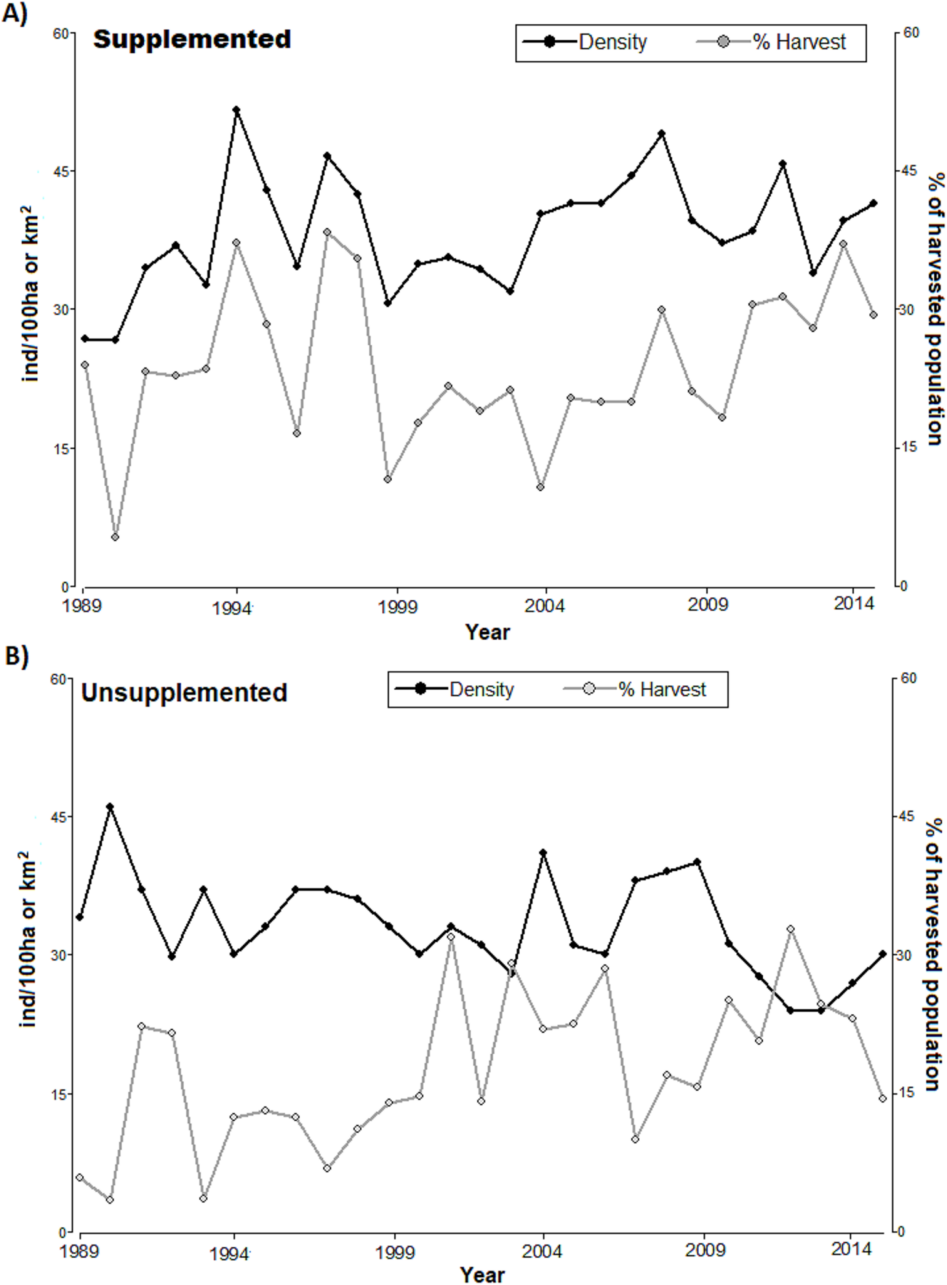
Annual population density (black line, ind/km^2^) and annual harvest (grey line, % of harvested population) for (A) supplemented and (B) unsupplemented populations, during the study period.

The densities in LM population increased since 1989 and ranged around 40 ind/km^2^ with considerable peaks (reaching above 50 ind/km^2^ in 1995, Fig. 1). LQ population ranged consistently between 30 and 40 ind/km^2^ overall, until 2009 with a consistent declining trend at the end of the study period, although with considerable peaks (e.g. high 2009), reaching the lowest level in 2014 (slightly above 20 ind/km^2^). With the exception of the first 4 years, the density was consistently higher in LM than in LQ population (Table 1; Fig. 1). Overall, densities were lower in LQ population, and population changes in both populations did not show any regular and/or cyclical pattern but marked ‘saw-tooth-like’ curves.

The exponential growth rate achieved values close to 0 in both populations (LQ = 0 y LM = 0.05); however, annual variations were significant, with positive and negative values (Table 1). LM population presented higher birth, recruitment survival rates, and proportions of calves and yearlings; while the proportions of females and adults were higher in LQ population (Table 1). In addition, the proportion of the population that was annually harvested and the harvest density were higher in LM population, with relevant annual variations in both study areas (Table 2; Fig. 1). Both populations presented sex ratios balanced slightly favourably to females.

**Table 2:** Total number of animals hunted from each population per year, theoretical maximum sustainable yield; MSYt) and differences between real values and those under the assumption of MSYt, separately for each management system or population (LQ, without supplemental feeding; LM, with supplemental feeding).

Year	LQ					LM			
	*N*° Total hunted	% of hunted MSYt	Sex-age class	Differences in real value and %	*N* Total hunted	% of hunted MSYt	Sex-age class	Differences in real value and %	
1989	138	40.00	50/20/68	−207/−60%	37	63.79	12/10/15	−21/−36%	
1990	107	31.01	37/16/54	−238/−69%	11	18.97	10/0/1	−47/−81%	
1991	564	163.48	103/33/428	219/63%	54	93.10	20/4/30	−47 −7%	
1992	444	128.70	115/33/296	99/29%	69	118.97	28/9/32	11/19%	
1993	91	26.38	46/5/40	−254/−74%	78	134.48	42/8/28	20/34%	
1994	256	74.20	50/20/186	−89/−26%	108	186.21	49/12/47	50/86%	
1995	298	86.38	98/70/130	−47/−14%	130	224.14	54/30/46	72/124%	
1996	315	91.30	120/28/167	−30/−9%	62	106.90	38/12/12	4/7%	
1997	173	50.14	113/10/50	−172/−50%	118	203.45	52/22/44	60/103%	
1998	275	79.71	161/20/94	−70/−20%	148	255.17	63/28/57	90/155%	
1999	317	91.88	136/31/150	−28/−8%	42	72.41	19/2/21	−16/−28%	
2000	302	87.54	117/26/159	−43/−12%	47	81.03	13/7/27	−11/−19%	
2001	723	209.57	30/169/524	378/109%	66	113.79	27/6/33	8/14%	
2002	301	87.25	73/60/168	−44/−13%	59	101.72	31/9/19	1/2%	
2003	558	161.74	132/118/308	213/62%	64	110.34	29/9/26	6/10%	
2004	619	179.42	155/127/337	274/79%	29	50.00	26/1/2	−29/−50%	
2005	478	138.55	83/130/265	133/39%	72	124.14	33/11/28	14/24%	
2006	588	170.43	142/128/318	243/70%	72	124.14	27/14/31	14/24%	
2007	259	75.07	85/34/140	−86/−25%	73	125.86	32/13/28	15/26%	
2008	454	131.59	83/126/245	109/32%	118	203.45	42/23/53	60/103%	
2009	430	124.64	119/104/207	85/25%	91	156.90	38/14/39	33/57%	
2010	537	155.65	181/155/201	192/56%	63	108.62	19/23/21	5/9%	
2011	392	113.62	134/67/191	47/14%	100	172.41	42/18/40	42/72%	
2012	539	156.23	145/152/242	194/56%	107	184.48	45/30/32	49/84%	
2013	407	117.97	81/77/249	62/18%	114	196.55	54/22/38	56/97%	
2014	429	124.35	134/88/207	84/24%	112	193.10	38/27/47	54/93%	
2015	297	86.09	101/28/168	−48/−14%	108	186.21	38/26/44	50/86%	
Annual Mean	381	110.43	105/69/207	36/year 10.4%	79	136.21	34/14/31	21.7/year 37.4%	
Total	10,291		2,824/1,875/5,592	976	2152		921/390/841	586	

**Note**:

MSYt is achieved at a population density and size of 16.32 and 19.73 ind/km^2^ and 1,136.2 and 171.2 ind. for LQ and LM, in K_50%_ respectively. MSYt values assume 5% natural mortality (Benton, Grant & Clutton-Brock, 1995). MSYt value per year was 345 for LQ (9,315 for all period) and 58 for KM (1,566 for all period). Sex-age class correspond to stags/calves/hinds, respectively.

Regarding harvesting, the extraction rates followed irregular patterns (see Fig. 1), alternating between periods of high and low hunting. The proportion of harvested tended to increase in LQ population during the last period of study resulting in a reduction of density (significant negative relationship between the annual proportion of harvest and density along the study period in LQ population, Beta = −0.35, *F* = 12.9, *p* < 0.01). There was an association between harvest density and population density in LM population, especially during 1989–1999 (*p* < 0.05, *T* = 2.21); however, low extraction rates during 2000-2007 led to an irregular, but increasing, density pattern.

### Harvesting parameters in relation to the MSYt value

The linear model evaluating the relationships between the exponential annual population growth rates and densities calculated for both populations for the period 1989–2015 evidenced a decrease in population growth at high densities in both situations, whether artificial feeding was provided or not (*F* = 30, *p* < 0.001) (Table 3), while age and sex class specific extraction rates, as well as the interactions between area and the other parameters (including density) were not significant.

**Table 3:** Linear Model, to explain exponential growth rate.

Variable	Df	*F*-value	*p*-value	Coefficient ± E.S.
Intercept	1	20.2	0.001	0.7 ± 0.21
Area	1	0.07	n.s.	0.026 ± 0.31
Density-1	1	30.3	0.001	−2.05 ± 0.44
Male hunted/Male total	1	1.13	n.s.	0.13 ± 0.07
Female hunted/Female total	1	0.05	n.s.	0.44 ± 0.6
Age class 1 hunted (<1 year)/age class 1 total	1	0.01	n.s.	0.44 ± 0.35
Age class 2 hunted (>1 year)/age class 2 total	1	0.2	n.s.	−0.79 ± 0.8
Area*Density-1	1	0.01	n.s.	0.07 ± 0.73
Area * % Male hunted	1	0.38	n.s.	0.37 ± 0.6
Area * % Female hunted	1	0.07	n.s.	−0.48 ± 1.78
Area * % Age class 1	1	3.02	n.s.	−0.92 ± 0.53
Area * % Age class 2	1	0.01	n.s.	0.23 ± 2.83

**Note:**

*F*, *p*-value and coefficients of the variables included in the Linear Model, to explain exponential growth rate. df show the degree of freedom of the numerator.

**Figure 2:**
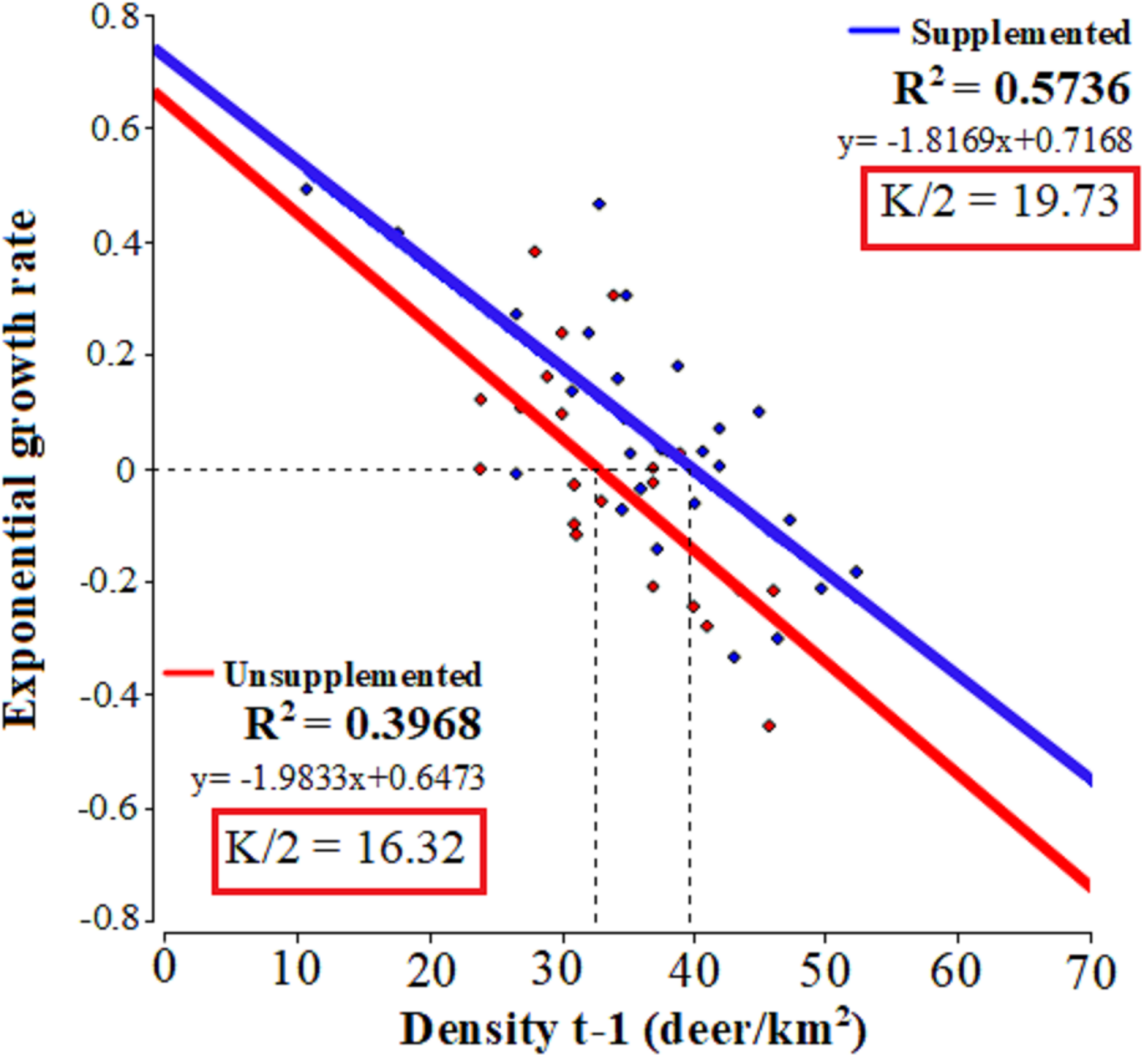
Tests of statistical density dependence. The tests of statistical density dependence derived from the logistic growth model, separately for each study population (supplemented = red and unsupplemented = blue).

The linear regressions fitted to relate the exponential growth rate and population density in each population (Fig. 2) evidenced that both populations presented significant linear adjustments (LQ: *F* = 13.15, *p* = 0.0017; LM: *F* = 33.62, *p* = 0.001; *R*^2^ = 0.57 and 0.39, respectively) with similar slopes 0.72 Lm (CI [0.47–0.96]) and 0.65 LQ (CI [0.26–0.98]). In LM population, the test of statistical density dependence established *K* at 39.45 deer/km², while for LQ population it was 32.64 deer/km². These values are similar to the average densities occurring during the study period: 37.9 and 32.9 deer/km² for LM and LQ, respectively. Therefore, the range of *I*-carrying capacity assuming 50 of *K* value was between 19.73 deer/km^2^ and 16.32 deer/km^2^ for LM and LQ populations, respectively.

**Figure 3:**
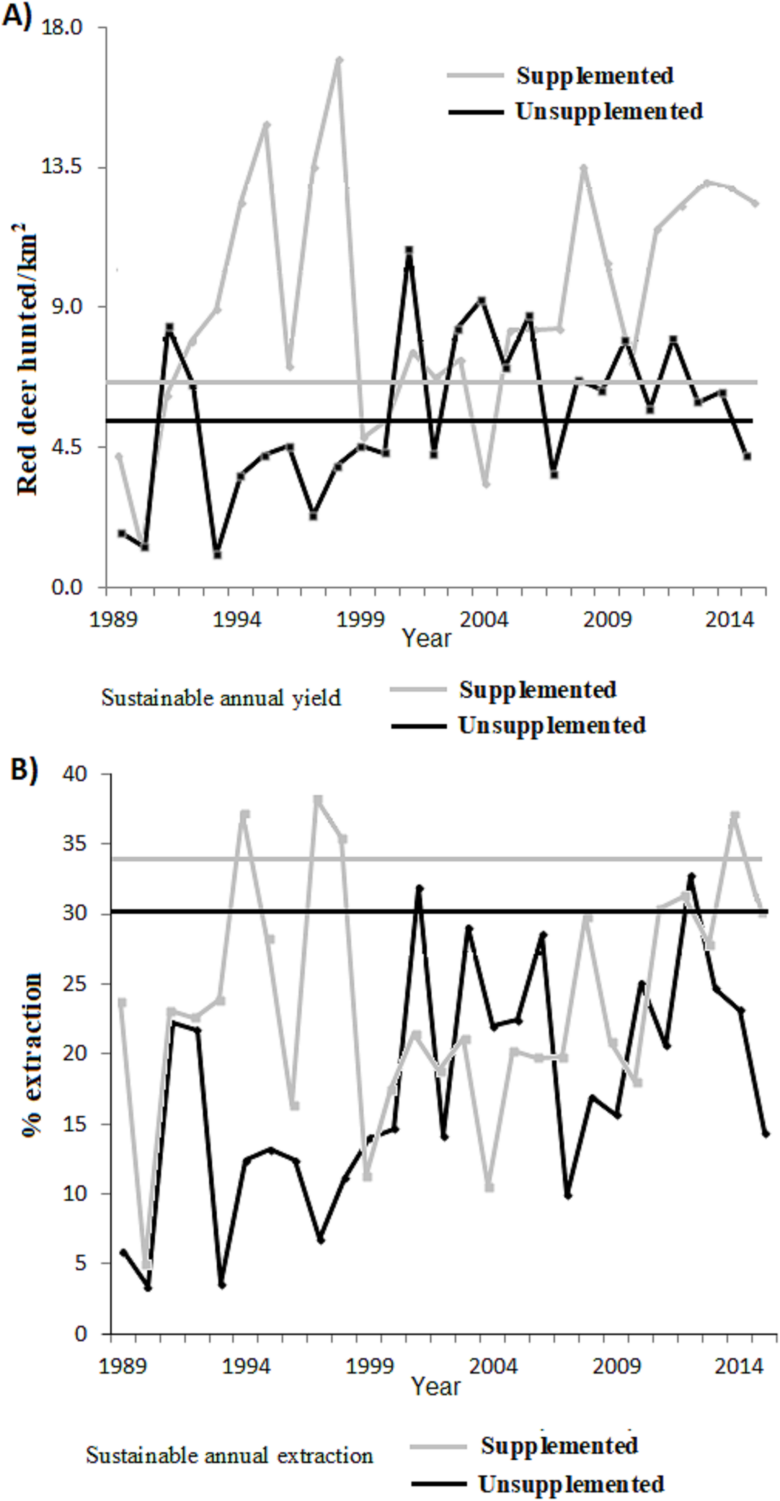
(A) Annual number of animals harvested depending on the surface area (n° ind/km^2^) and (B) proportion of extraction with respect to the total population for the supplemented (grey line) and unsupplemented estates (black line). For comparison, horizontal lines indicate the predicted value in a situation of MSYt (dashed lines).

Figure 3 plots the harvested density and the proportion harvested for both populations, indicating as a reference their values at MSY (for K_50%_ according to our previous estimations of*I*theoretical values). Table 2 shows the total number and Table 4 the proportion harvested in each population. The total number of animals hunted was slightly higher in relation to what was expected under an MSY assumption for both populations. In the case of the extraction rates, the real observed numbers were below the percentage of sustainable extraction for the whole study period, except at 2 years in the case of LM and at 4 years in LQ (Table 2).

**Table 4:** Percentage of animals hunted from each population per year, theoretical maximum sustainable yield (MSYt), and differences between real percentages and those under the assumption of MSYt, separately for each management system or population (LQ, without supplemental feeding; LM, with supplemental feeding). MSYt values assume 5% natural mortality.

Year	LQ			LM		
	% Harvest	MSYt	Differences	% Harvest	MSYt	Differences
1989	6.1	30.74	−24.6	23.8	34.04	−10.2
1990	3.4		−27.3	4.7		−29.3
1991	22.2		−8.5	23.2		−10.8
1992	21.7		−9.1	22.7		−11.3
1993	3.6		−27.1	23.9		−10.1
1994	12.4		−18.3	37.6		3.6
1995	13.2		−17.5	28.4		−5.6
1996	12.4		−18.3	16.3		−17.7
1997	6.8		−23.9	38.6		4.6
1998	11.1		−19.6	35.8		1.8
1999	14.1		−16.6	11.2		−22.8
2000	14.7		−16.1	17.5		−16.5
2001	31.9		1.2	21.5		−12.5
2002	14.1		−16.6	18.8		−15.2
2003	29.1		−1.6	21.2		−12.8
2004	22.1		−8.6	10.3		−23.7
2005	22.5		−8.2	20.3		−13.7
2006	28.6		−2.1	19.8		−14.2
2007	9.9		−20.8	19.9		−14.1
2008	17.1		−13.6	30.1		−3.9
2009	15.7		−15.1	20.9		−13.1
2010	25.1		−5.6	18.1		−15.9
2011	20.6		−10.1	30.7		−3.3
2012	32.7		1.9	31.6		−2.4
2013	24.7		−6.1	28.1		−5.9
2014	23.1		−7.6	37.5		3.5
2015	14.4		−16.3	31.1		−2.9
Anual mean	17.5	30.74	**−13.31**	23.8	34.04	**−10.2**

Overall, an average of an extra 36 deer per year were hunted (0.52 deer annually hunted/km^2^) from LQ population compared with a sustainable MSY situation (345 individuals, 5.03 hunted/km^2^). This represents 10.4% more hunting bag compared to MSY. On the other hand, an extra 21.7 deer were hunted per year (2.5 hunted/km^2^) from LM population compared with a sustained MSY situation (58 individual, 6.68 hunted/km^2^), which represents 37.4% more hunting bag compared to (artificial) MSY. During the total study period (27 years) 976 and 586 fewer deer would have been hunted under an MSY situation for LQ and LM respectively (see Table 2). In terms of average extraction rates, a 13.3% and 10.21% lower proportion of the population would have been extracted compared to the MSY situation in LQ and LM populations, respectively (Table 4). In other words, the extraction rate would be 1.75 and 1.43 times higher under an MSY situation, for LQ and LM populations, respectively.

## Discussion

We have shown using two high density red deer populations under contrasted management schemes, that is with and without artificial feeding that (i) the relative rate of increase per individual similarly decreases linearly (similar slopes) with population density (under the logistic equation). Therefore, density dependence is still and similarly evidenced when supplementary food is provided generously all the year round. However, equilibrium theoretical population density, *N*, is increased by the effects of supplemental feeding on population productivity (see Rodriguez-Hidalgo et al. (2010), Yoccoz et al. (2001) for small mammals), even when natural food availability or habitat quality was apparently lower (Fig. 2). Secondly (ii) the unsupplemented population provided reference values to implement sustainable management of red deer populations adaptively in our study region. Altogether (iii) this study reveals the importance of monitoring big-game population dynamics from a long-term perspective, allowing the incorporation of adaptive management to decision taking. In this case, according to Williams (2011), adaptive management is defined as a learning-based process involving the fundamental features of learning (monitoring of red deer population dynamics through time) and adaptation (the adjustment of management (e.g. harvest) through time based on this learning). Current management schemes of big game in Europe normally lack proactive policies based on the understanding of population dynamics (Vicente et al., 2019), which is essential to make ecological and socio-economic aspects compatible when exploiting the resource. Long-term hunting strategies that are not proactive may lead to high population densities and undesirable overabundance situations, causing adverse effects on the population itself as well as on other species and even on the economy as the use of the resource may become unsustainable (Carpio, Apollonio & Acevedo, 2021; Valente et al., 2020).

### Density dependence and management

The average deer densities of the study populations might be considered high in the context of the values described in the international scientific literature, where large range of values exist for this plastic species even locally (Apollonio, Andersen & Putman, 2010), for example 3 deer/km^2^ in Sweden (Jarnemo, 2008), 3.7 deer/km^2^ in Norway (Mysterud et al., 2007), 8.1 deer/km^2^ in Scotland (Pérez-Barbería, Hooper & Gordon, 2013) or 9.6 deer/km^2^ in the Czech Republic (Prokesová, Barancekova & HoMolka, 2006). Although, Buckland et al. (1996) showed densities between 20 and 34 deer km^2^ in Scottish red deer populations. It should be remembered that Central European literature generally considers that much lower densities than those described here (e.g. densities above 4 deer/km^2^) are not compatible with other activities of agricultural and forestry production and may have adverse effects on the conservation of the natural environment (Gill, 1992). Similar high ranges of densities have been found in other studies in Mediterranean ecosystems and areas with a temperate climate, for example up to 40 deer/km^2^ in Portugal (Silva et al., 2014), >30 deer/km^2^ in other areas of Spain (Perea, Girardello & San Miguel, 2014) or 26 deer/km^2^ in Italy (Casula et al., 2009), 20–34 deer/km^2^ in Scotland (Buckland et al., 1996). These values indicate that the carrying capacity of red deer in Mediterranean and temperate oceanic environments may present relatively high values, compared to northern or continental latitudes. Climatic conditions and the quality of the food available (e.g. Real Bioclimatic Index, Martínez-Jaúregui et al., 2009) are adverse in the summer in Southern Europe due to drought (with a second minimum in winter), showing significant interannual variability. However, the vegetation productivity curve in our study latitudes varies little over the year due to the presence of evergreen trees and shrub species (Gazol et al., 2018), which contrasts with the Northern and Central latitudes of Europe, where hard winter conditions limit winter survival, determining lower average carrying capacities (Martínez-Jaúregui et al., 2009). However, NDVI varies considerably among plant species, droughts, and the location of the studied forests (Gazol et al., 2018). These authors also indicated than NVDI data are less sensitive metrics of forest resilience to drought than other metrics as TRWi (absolutely dated ring-width indices).

The relative rate of increase per individual decreased linearly with population density in both study populations. However, equilibrium population density, *K*, is increased by supplemental feeding. This happened even when natural conditions or habitat quality apparently was lower in the supplemented population (Milner et al., 2014). López-Montoya, Moro & Azorit (2017) detected that density-dependence may have a stronger effect on growth than hunting or climatic conditions in populations which are closer to carrying capacity (these aspects are being addressed for these two populations in a separate article). A high population density experienced early in life may cause a reduction in subsequent development, thus affecting antlers size over time (Torres-Porras, Carranza & Perez-Gonzalez, 2009; Pérez et al., 2011), which may be detrimental in terms of the objectives of hunting. Miranda et al. (2015) showed that supplemental feeding provided to ungulates in LM led to increased browsing on plant species whose nutritional composition complemented that of the supplement provided. In our study populations, Acevedo et al. (2008) reported high levels of browsing in both study sites (0.95 and 0.86 browsing indexes for highly palatable species, and 0.85 and 0.83 browsing indexes for species with reduced palatability, in LQ and LM populations, respectively), which are high according to previous studies (Morellet et al., 2001). In addition, aggregation at feeders may increase disease transmission (Barasona et al., 2017). Vicente et al. (2007) showed that supplemental feeding increases the body condition at the cost of increased host contact rates, which implied an ecological trade-off between acquiring resources and the concurrent exposure to mycobacterias (the causative agent of animal tuberculosis, in this case). Understanding these long-term complex links represents an important contribution to the planning and prioritising of population management actions.

We also verify that the neither the population growth rate nor its negative relationship with population density in both study populations was affected or mediated by the rates of harvesting different age and sex classes. This maybe result of the hunting strategies implemented in the study sites, where not specific plans intended population reduction by targeting specific sex by age classes.

### Harvest rates and proactive sustainable management

The high average densities (close to theoretical *K* values) only yielded 10.4% more hunting bag compared to the MSY in LQ population (this population can be considered as a reference for the natural carrying capacity in our study area). However, the disproportional increase in deer density to achieve such a limited hunting bag increase may in turn lead to negative impacts (see above). As for LM population, the hunting strategy yielded 30.4% more hunting bag compared to MSY due to very high densities (which peaked at up to 50 deer/km^2^) reached in a sub-optimal habitat for red deer. Therefore, this population cannot be considered a reference of natural circumstances. Theoretically, in LQ population, a density of 16.3 deer/km^2^ may allow extraction rates of up to 30.3% (MSY); however, the actual extraction rate (17.5%) is well below the MSY threshold. Previous studies (such as DeCalesta (2017) or Mihalik (2016)) showed similar situations in wild ungulate populations where the extraction rate is below the sustainable rate under high density situations, and where an increased effort in the harvest rate reduced deer density, thus reducing impacts on ecosystems, while improving deer health and hunter satisfaction (Ueno, Kaji & Saitoh, 2010), although this depends on hunter’s preferences (e.g. trophy, shoots, meat; see Heberlein, 2002).

Our harvest rates compare with the range described by previous authors (Kiffner et al., 2008; Saint-Andrieux et al., 2009; Apollonio, Andersen & Putman, 2010; Martínez & Martín, 2017). They respond to local situations in space, time and management (Milner et al., 2006); however, we evidenced that the significant changes in harvest rates were a consequence of reactive decision taking. Buckland et al. (1996) suggest that the female (hind) cull should be restricted to non-lactating adult (yeld) and immature hinds, reducing hind numbers so that there are just two hinds of age >1 for every stag of age >6 in the population, provided that the population is maintained well below the carrying capacity (roughly estimated at 1,400 deer for LQ; Millas-Prendergast & Martín-Fernández, 2015), which approaches the situation we described for LQ population (*K/2* population was estimated at about 1,268 deer). Proactive adaptive hunting management can maintain population levels within an optimum range, maximising the balance between forage resources and population productivity (Pérez-González et al., 2010; Rodriguez-Hidalgo et al., 2010; Herruzo & Martínez-Jauregui, 2013; Herruzo et al., 2016); that is when the population is at the optimal level, growth is high, and deer will be large (McCullough, 1979). For example Santín-Durán et al. (2004) showed a positive relationship between the intensity of parasite infections and population density of red deer in LQ, and Gómez et al. (2001), who showed the impact of red deer on vegetation, also recommended limiting the deer population in LQ. In this population, previous studies found that density was the most important factor explaining the variation in conception dates, with greater densities causing later conception dates (Peláez et al., 2017), as well as having a negative effect on antler quality (Peláez et al., 2018). In the same way, Santos et al. (2018) found a negative density-dependent response of deer body reserves when considering the combined effects of population density and dietary quality, especially in populations without a supplementary feeding regime (as in LQ). Perea, Girardello & San Miguel (2014) showed that some plant species were highly preferred and were not to be found in the areas where the deer were present in great densities (>30 ind/km^2^) for a long period of time (for example LM).

### Priorities for managing hunted red deer populations in Mediterranean areas

Density-mediated factors determined the population growth in these two situations of high red deer densities in a Mediterranean habitat, even when artificial feeding was provided. Our research has shown that, in these particular situations, an increase in the extraction rates (as is already happening in LQ) is needed.

### Management implications

The extraction rates reported here can be considered as indicative (rather than reference) values for the two prevalent hunting management regimes in the south-central Iberian Peninsula. Hunting plans must be based adaptively on population monitoring, thereby proactively preventing resource damage. For these purposes, a simple initial approach can be to fix sustainable hunting rates to mitigate the impacts of deer browsing. We must promote awareness of the problem among policy makers and managers, but also develop and apply available population monitoring tools, to be applied in proactive management plans, with active surveillance and the early detection of the consequences of overabundance. Adaptive management involves assessing the effectiveness of management actions in relation to the plan’s goals and objectives (Lyons et al., 2008).

## Supplemental Information

10.7717/peerj.10872/supp-1 Supplemental Information 1 Raw data.

## Additional Information and Declarations

### Data Availability

The following information was supplied regarding data availability: Data is available in Tables 2 and 3 and the Supplemental Files.
